# Unveiling the hidden connection: Investigating the relationship between shared leadership and missed nursing care

**DOI:** 10.1016/j.ijnss.2024.12.012

**Published:** 2024-12-17

**Authors:** Amal Diab Ghanem Atalla, Naglaa Abdelaziz Mahmoud Elseesy, Ayman Mohamed El-Ashry, Samia Mohamed Sobhi Mohamed, Ebaa Marwan Felemban, Abdulhafith Alharbi, Nervana Abdelrahman Saied Gheith, Sabrein Mahmoud khalifa khattab

**Affiliations:** a Department of Nursing Administration, Faculty of Nursing, Alexandria University, Alexandria, Egypt; b Department of Public Health Nursing, Faculty of Nursing, King Abdulaziz University, Jeddah, Al-Qurayyat, Saudi Arabia; c Department of Nursing, Collage of Applied Medical Sciences, Al-Qurayyat, Jouf University, Al-Qurayyat, Saudi Arabia; d Department of Psychiatric and Mental Health Nursing, College of Nursing, University of Hail, Hail, Saudi Arabia; e Department of Nursing Administration, Faculty of Nursing, Mansoura University, Mansoura, Egypt

**Keywords:** Missed nursing care, Nurses, Nursing administration research, Shared leadership

## Abstract

**Objectives:**

This study examined the levels of shared leadership and missed nursing care and their relationship among nurses in Egypt.

**Methods:**

A cross-sectional survey was conducted. From February to April 2024, 340 nurses worked in all inpatient care units at Alexandria Main University Hospital. The Shared Leadership and Missed Nursing Care Survey was used for evaluation.

**Results:**

The overall score of shared leadership was (72.62 ± 4.30), which imitates a high perception of shared leadership among nurses; the dimension of delegation achieved the highest average mean score (3.66 ± 0.26), followed by collaboration (3.64 ± 0.31), the vision dimension scored the lowest mean score (3.59 ± 0.33). The elements of missed nursing care had a total score of (46.72 ± 5.69), and the dimension of secondary care achieved the highest average mean score (3.74 ± 0.31). The reasons for missed nursing care had a total mean score of (22.40 ± 1.59), and the dimension of labor resources achieved the highest average mean score (3.20 ± 0.22). Male nurses, less than 30 years old, married, held a bachelor’s degree in nursing sciences, less than five years of experience in the nursing profession, and less than five years of experience in the current working unit had higher total scores of the shared leadership and lower total scores of missed nursing care (*P* < 0.001). A negative correlation existed between shared leadership and two dimentisons (the elements of nursing care [*r* = −0.383], the reasons for missing nursing care [*r* = −0.047]) (*P* < 0.001).

**Conclusions:**

The study’s findings can help as a theoretical underpinning for nursing leaders to stand in an environment that reduces the incidence of missed nursing care by encouraging teamwork, responsibility, workload management, and empowerment among nurses.

## What is known?

• In healthcare systems, neglected nurse care and shared leadership is a major worldwide issue that requires attention. Shared leadership behaviors are positively correlated with safer healthcare practices. Missed nursing care can result in unfavorable patient outcomes, jeopardized safety, escalated expenses, and reduced satisfaction.
• Research on shared leadership implementation in healthcare settings is ongoing to identify the obstacles and enablers of this practice and assess its long-term effects on patient outcomes, healthcare quality, and worker satisfaction.

## What is new?

• Recent research has highlighted the importance of shared leadership in addressing complex healthcare challenges. It emphasizes the need for shared decision-making, coordination, and information sharing among healthcare teams.
• Based on the study findings, we highlight the importance of efforts being made to develop standardized measures and metrics to assess missed nursing care, allowing for better monitoring and benchmarking across healthcare organizations.

## 1 Introduction

Healthcare delivery systems now have to deal with tremendous change and the quick development of new inventions, information technology, and communication networks. The organizational structure and methods of healthcare have changed more quickly and dramatically, and as a result of this evolution, leadership has been reformed into a critical component for the upkeep and advancement of the contemporary healthcare system [1].

Shared leadership fosters individual collaboration in learning, allows for creative expression, and guarantees follower empowerment, all crucial elements for modern healthcare advancement [2]. Historically, a leader’s identity has been linked to a formal leadership role or a hierarchical position despite the growing trend to view leadership as a distributed and collaborative activity [3]. The idea that leadership is an emergent dynamic process “shaped in and by everyday practices and the environment” is gaining traction in leadership studies as a multimodal broad accomplishment in which participants' exact expressions, gestures, object usage, and arrangement are examined as real-time interactional phenomena using transcripts of naturalistic interactions [4].

Zhu et al. [5] have identified three key characteristics of shared leadership: the division of leadership tasks and influence among team members, an emergent team phenomenon, and lateral influence among peers. The first source of leadership influence is a peer-to-peer lateral influence in cooperative work. Secondly, the unit of analysis is a phenomenon that emerged as a team. The primary focus of the study is the team members. In contrast to traditional leadership, which is a phenomenon created by a single individual, shared leadership is a phenomenon that characterizes leadership as an emerging team trait of numerous team members [6]. Thirdly, the distribution of influence and leadership duties across team members highlights the division of duties and influence inside the team leadership hierarchy. In contrast to traditional leadership systems, which centralize leadership around one individual, shared leadership essentially entails the idea that leadership influence is “broadly distributed” across various team members [5,6]. Shared leadership is a valuable approach that can be used in Egypt to improve productivity, stimulate creativity, and increase job satisfaction. Egypt’s vision for addressing the difficulties of the twenty-first century concludes that one of the components required to address these challenges can be shared leadership. Organizations face increasing issues due to demographic shifts, rapid technical advancements, and global economic volatility [6].

Shared leadership is linked to better health services, such as higher employee satisfaction, lower attrition, and higher-quality care. Team members are seen to provide more resources, share more knowledge, and be more committed to the group when shared leadership is practiced, which results in much better levels of trust and respect. In 2023, Beiboer et al. [[7], [8], [9]] showed that enhancing the clinical leadership abilities of nurses could decrease missed care and improve teamwork. Missing nursing care is a crucial metric for assessing the standard of nursing care and guaranteeing patient safety. Kalisch first recognized the phenomena of missed nursing care [10]. It is believed to be an indicator closely linked to the caliber of care and patient outcomes [11,12]. Missed nursing care is defined as nursing care that is not and is concerned with the quality of care, according to the International Hospital Outcomes Research Consortium (IHORC) [13]. Required nursing care that is postponed, rendered in part, or not rendered at all is called missed nursing care. It falls under the subcategory of omission errors.

The health sector’s complicated structure augments the necessity for shared leadership, the information technology industry’s rapid advancements, and the pressure on workers to boost productivity [14]. Across all healthcare systems, nurses constitute the majority of healthcare workers. One crucial indicator of the caliber of nursing care and patient safety is the rise in missed nursing care. Missed nursing care can be affected by leadership style; one unique leadership strategy that may have an impact on how formal and informal leaders affect nursing care that is neglected is shared leadership. It is crucial to investigate the relationship between missed nursing care and shared leadership, as opposed to more conventional hierarchical leadership styles [9].

In addition, organizations, nurses, and patients might all suffer from inadequate nursing care [9,15]. There is no distinct relationship between the absence of nursing care and ethical leadership [16]. However, a cross-sectional study in Iran discovered a strong negative correlation between perceived ethical leadership and neglected nursing care [17]. Research on the relationship between missed nursing care and shared leadership is scarce. Thus, research in this field is essential.

Shared leadership is a useful approach that can be used in Egypt to improve productivity, stimulate creativity, and increase job satisfaction [18]. Healthcare professionals’ stronger institutional and professional identities strongly correlate with shared leadership [19]. Any component of necessary patient care that is skipped or delayed is referred to as “missed nursing care,” it has been associated with several unfavorable patient outcomes [20].

This study explored the relationship between shared leadership and missed nursing care in the Egyptian healthcare system, where nursing shortages and high workloads are prevalent. It highlights the importance of shared leadership in empowering nurses and enhancing patient-centered care. Given the focus on interprofessional collaboration in Egypt, understanding this connection may help organizational leaders address the factors causing nurses to miss care opportunities. The research question centers on how nurses perceive the link between shared leadership and missed nursing care. This investigation aimed to inform strategies for improving nursing practice and patient outcomes.

## 2 Methods

### 2.1 Study setting and participants

A cross-sectional descriptive design was adopted. The study was carried out in an Egyptian hospital with 220 beds in total capacity. This covered all inpatient medical and surgical care units and ICUs (*n* = 16). These included: 1) medical units (*n* = 8), which included obstetric, pediatric, neurosurgical, dialysis, orthopedic, and poison units; 2) surgical units (*n* = 3), which included general surgical and operation units; and 3) ICUs (*n* = 5), which included general, neonatal, pediatric, coronary care, and emergency units. This hospital began taking significant action to meet the patient safety requirements set forth by the General Authority for Health Accreditation and Regulation (GAHAR). Situated in a vast catchment area, this hospital is among the biggest in Egypt and the Middle East, catering to a considerable populace. It is regularly used by hospitals in neighboring countries and many nationwide hospitals as a tertiary referral facility. Therefore, improved nurse career planning and retention can lead to better patient outcomes and higher-quality healthcare services, which benefit the community.

A convenient sampling method (a non-probability sampling methodology in which researchers choose participants based on availability and ease of access) was conducted. The study covered every intended nursing population. Nurses who had worked in the aforementioned units for at least one year made up the study (*n* = 300) to familiarize them with administrative rules, policies, and regulations in addition to the hospital system. The nurses also had to be present when the information was being gathered. Nurses who met the following requirements were chosen to participate in the study: 1) they needed to work in pre-selected environments; 2) they needed to have been in the working unit for at least a year; and 3) to offer nursing care to patients directly.

### 2.2 Instrument

#### 2.2.1 Sociodemographic information questionnaire

This questionnaire gathered information on demographic characteristics, including years of experience in nursing, years of experience in the work units, gender, age, and educational qualifications.

#### 2.2.2 Shared Leadership Questionnaire

Brussow created it to gauge nurses’ belief in shared leadership. It has 20 items broken down into the four primary common leadership areas. They are separated into four domains: delegation (6 items), culture (5 items), vision (4 items), and cooperation (5 items) [21]. A 5-point Likert scale, with “1” denoting strongly disagree and “5” denoting strongly agree, will be used for participant responses. The total means score range for the Shared Leadership Questionnaire is (20–100). For the current study, Cronbach’s *α* coefﬁcient was 0.90.

#### 2.2.3 The Missed Nursing Care Survey (MISSCARE survey)

This questionnaire was developed by Kalisch & Williams (2009) [22] and validated by Hosseini et al. (2022) [23]. This survey is divided into two parts. Part A namely: elements of missed nursing care, has 24 items and three dimensions namely: necessary care (13 items), supportive care (5 items), and secondary care (6 items) answered on a 5-point Likert scale, with “5” being always absent and “1” never missing [22]. Part B namely: reasons for missed care consists of 17 questions and five dimensions concerning the reasons for not receiving nursing namely: labor resource (3 items), material resource (3 items), communication (4 items), responsibility (4 items), and unpredictable situations (3 items). Part B questions are graded on a 4-point Likert scale, with “4” representing importance and “1” denoting not a cause to miss nursing care. Greater scores correspond to greater volumes of neglected care or a more important cause of neglected nursing care tasks. The total score was calculated by recording the responses. A higher score denotes a higher level of unmet nursing care (Part A) and a more substantial reason for that lack of care (Part B). Both sections are scored independently, with part A providing an overall measure of care missed, while part B identifies contributing factors. There isn’t a single overall score, but the responses in each section help to understand both the frequency and the reasons for missed care. This system allows for a separate analysis of missed care types (from A) and the barriers to providing care (from B), offering a comprehensive view of nursing care gaps in a clinical setting. Pearson correlation coefficient yielded a value of 0.933 on part A and 0.910 on part B [23].

#### 2.2.4 Data quality control technique

Rewritten interpreted, and translated the English version of the questionnaire into Arabic and confirmed their face validity. Three experts in the field of the study assessed face and content validity and guided things like relevance, the accuracy of interpreted questions, and clarity of content. The experts had backgrounds in the field of the study and vast knowledge of psychometrics and research techniques.

Their remarks were thoughtful to ensure accuracy. The researchers utilized an exploratory factor analysis to evaluate the validity of the Shared Leadership Questionnaire. Before and after varimax rotation, the loading score varied from 0.512 to 0.966 and 0.544 to 0.951, respectively. This was more significant than 0.35 and accounted for 82.15% of the variance in total. With a sampling adequacy of 0.923, according to Kaiser-Meyer-Olkin, the data was deemed appropriate for factor analysis. Furthermore, Bartlett's test of sphericity achieved statistical significance (*P* < 0.001), confirming the correlation matrix's factor capacity. As a result, the questionnaire's items were kept. The researchers utilized an exploratory factor analysis to evaluate the validity of the MISSCARE Survey. Before and after varimax rotation, the loading score varied from 0.662 to 0.912 and 0.598 to 0.982, respectively. Greater than 0.45, this explained 79.45% of the variation in total. With a sampling adequacy of 0.923, according to Kaiser-Meyer-Olkin, the data was deemed appropriate for factor analysis. Furthermore, Bartlett’s test of sphericity achieved statistical significance (*P* < 0.001), confirming the correlation matrix's factor capacity. As a result, the questionnaire’s items were kept.

Ten percent of the 340 nurses (*n* = 34) authorized the pilot research to defend the goods’ simplicity and practicality by identifying potential roadblocks and issues during data collection. Nothing needed to be altered. The study did not include those who took part in the pilot study. The researchers checked the questionnaires for accuracy and completeness.

### 2.3 Ethical considerations

The research ethics committee of Alexandria University’s Faculty of Nursing approved the study protocol (Serial Number: AU-20-8-246). The purpose of the study was communicated to nurses before they gave their agreement. Each questionnaire was given a code number to protect confidentiality and identity. As promised to nurses, data use was limited to research. The ability to withdraw from the study has been confirmed.

### 2.4 Data collection

Personalized copies of the study questionnaires were given to the participants. The researchers gave a hand-delivered questionnaire to each nurse and then collected the completed forms later. After explaining the study’s goal to each nurse individually for 2 min, the nurses were asked to return it to the researcher. These questionnaires were completed in front of the researcher to ensure the respondents’ objectivity, the integrity of their thoughts, and the completion of all questions. Because they were connected to specific working units, monitoring the delivery and gathering to guarantee the highest response rate was easy. In exchange for their participation, participants received little snacks. The questions should take 15–20 min to complete. The data collection period spanned three months, from February to April 2024. All of the nurses’ questions were answered, and explanations were given.

### 2.5 Data analysis

Data analysis was conducted using SPSS version 23.0. Mean and standard deviation were used to analyze the data. Continuous values were described by mean and standard deviation, while categorical values were described by frequency and percentage. The ANOVA test is utilized when comparing the means of three or more groups. It can assist in determining whether there are notable variations in the amounts of nursing care missed among various levels of shared leadership approaches. Following an ANOVA, the Scheffé test is a post hoc analysis to determine which group means vary. The Scheffé test can identify which leadership styles are associated with higher or lower nursing care rates missed if the ANOVA shows significant differences. To determine whether there is a statistically significant difference in the amount of nursing care missed between two distinct leadership styles, the student’s *t*-test compares the means of two groups. The degree of shared leadership and the amount of missed nursing care are evaluated using Spearman’s correlation.

## 3 Results

### 3.1 Participants characteristics

The study’s results show that the majority of the nurses were between 30 to less than 40 years old (61.5%), female (77.4%), single (77.4%), and graduated from the technical nursing institute (66.2%). Most nurses had 5 to less than 10 years of experience in nursing (54.7%) and their current working unit (54.7%).

### 3.2 Overall status of shared leadership and missed care among nurses

Table 1 presents that the overall score of shared leadership was (72.62 ± 4.30), which imitates a high perception of shared leadership among nurses. Explicitly, the highest reason’s mean score referred to the dimension of delegation (3.66 ± 0.26), while the vision dimension scored the lowest mean score of shared leadership (3.59 ± 0.33). Concerning part A of the MISSCARE survey, which refers to the elements of missed nursing care, Table 1 illustrates that it had an overall score of (87.44 ± 7.97). Exactly, the highest reason’s mean score was about the dimension of secondary care (3.74 ± 0.31). Regarding part B of the MISSCARE survey, which refers to the reasons for missed nursing care, Table 1 shows an overall score of (54.05 ± 3.42). Precisely, the highest reason’s mean score was about the dimension of labor resources (3.20 ± 0.22), the lowest mean scores of the reasons for missed nursing care were equally related to both dimensions namely: responsibility and unpredictable situations (3.13 ± 0.31).

**Table 1:** The score of the shared leadership and nursing missed care among nurses (*n* = 340).

Dimensions	Total score	Mean score
Shared leadership		
Collaboration	18.23 ± 1.55	3.64 ± 0.31
Vision	14.37 ± 1.35	3.59 ± 0.33
Delegation	22.00 ± 1.61	3.66 ± 0.26
Culture	18.00 ± 1.40	3.60 ± 0.28
Overall	72.62 ± 4.30	3.63 ± 0.21
Nursing missed care		
Elements of missed nursing care		
Necessary care	46.72 ± 5.69	3.59 ± 0.43
Supportive care	18.24 ± 1.58	3.64 ± 0.31
Secondary care	22.47 ± 1.87	3.74 ± 0.31
Overall	87.44 ± 7.97	3.64 ± 0.33
Reasons for missed care		
Labor resources	22.40 ± 1.59	3.20 ± 0.22
Communication	12.67 ± 1.13	3.16 ± 0.28
Responsibility	9.39 ± 0.93	3.13 ± 0.31
Unpredictable situations	9.59 ± 0.93	3.13 ± 0.31
Overall	54.05 ± 3.42	3.17 ± 0.20

*Note*: Data are *Mean* ± *SD*.

### 3.3 The inﬂuence of sociodemographic variables on the shared leadership and missed care

There were statistically significant differences in the total score of the shared leadership based on all sociodemographic characteristics (*P* < 0.001). Also, there were statistically differences in the total score of the elements of missed nursing care (part A of the MISSCARE survey) based on nurses’ sociodemographic characteristics, namely marital status, educational qualifications, and years of experience in the nursing profession. In addition, there were statistically differences in the total score of the reasons for missed nursing care (part B of the MISSCARE survey) based on nurses’ sociodemographic characteristics, namely, gender and years of experience in the nursing profession and the current working unit (*P* < 0.001). Shared leadership was higher among nurses aged from 20 to less than 30 years old and married nurses with a bachelor’s degree in nursing science (*P* < 0.001). Both elements of and reasons for missed nursing care were higher among female single nurses (*P* < 0.001). The reasons for missed nursing care were higher among nurses who had from 5 to less than 10 years of experience in the nursing profession (since graduation) (Table 2).

**Table 2:** The score of the shared leadership and missed nursing care among nurses with different characteristics (*n* = 340).

Characteristics	*n* (%)	Shared leadership	*t/F*	*P*	Elements of missed nursing care	*t/F*	*P*	Reason for missed nursing care	*t/F*	*P*
Gender										
Male	77 (22.6)	74.56 ± 5.50	4.612	<0.001	84.81 ± 7.69	3.351	0.001	53.38 ± 4.15	1.995	0.047
Female	263 (77.4)	72.06 ± 3.72			88.22 ± 7.91			54.26 ± 3.16		
Age (years)										
20–< 30	48 (14.1)	74.44 ± 6.64	8.182	<0.001	87.17 ± 11.73	0.892	0.445	51.35 ± 3.26	16.520	<0.001
30–< 40	209 (61.5)	72.89 ± 3.88			87.06 ± 8.31			54.16 ± 3.22		
40–<50	83 (24.4)	71.01 ± 2.22			89.15 ± 3.06			54.97 ± 2.38		
Marital status										
Married	21 (6.2)	76.48 ± 4.50	16.427	<0.001	78.76 ± 10.57	12.985	<0.001	52.00 ± 2.00	5.173	0.002
Single	263 (77.3)	72.59 ± 4.27			88.54 ± 7.95			54.21 ± 3.50		
Widow	20 (5.9)	74.90 ± 1.65			83.45 ± 4.42			52.50 ± 0.69		
Divorced	36 (10.6)	69.33 ± 2.66			86.69 ± 2.05			55.00 ± 3.79		
Educational qualifications										
Bachelor’s degree in nursing sciences	86 (25.3)	75.60 ± 4.76	51.579	<0.001	81.33 ± 12.05	43.584	<0.001	53.88 ± 3.95	0.405	0.667
Technical nursing institute	225 (66.2)	72.09 ± 3.46			89.76 ± 4.42			54.17 ± 3.22		
Secondary nursing school diploma	29 (8.5)	67.90 ± 2.74			87.59 ± 3.88			53.69 ± 3.38		
Years of experience in the nursing profession (since graduation)										
1–<5	61 (17.9)	73.51 ± 6.53	14.027	<0.001	87.92 ± 10.71	3.372	0.019	52.11 ± 3.33	10.429	<0.001
5–<10	186 (54.7)	72.97 ± 3.55			86.48 ± 8.58			54.65 ± 2.93		
≥10	93 (27.4)	70.09 ± 1.855			88.23 ± 2.25			54.01 ± 3.51		
Years of experience in the current working unit										
1–<5	61 (17.9)	73.51 ± 6.53	7.130	<0.001	87.92 ± 10.71	2.258	0.081	52.11 ± 3.33	17.353	<0.001
5–<10	186 (54.7)	72.97 ± 3.55			86.48 ± 8.58			54.65 ± 2.93		
≥10	93 (26.4)	69.92 ± 1.75			89.06 ± 1.40			52.36 ± 1.93		

*Note*: Data are *Mean* ± *SD*.

### 3.4 The correlation between shared leadership and missed care among nurses

The study’s result illustrated a negative correlation (*r* = −0.383, *P* < 0.001) between the elements of nursing care missed and shared leadership, indicating that missed nursing care decreases as shared leadership increases. The correlation between the reasons for missing nursing care and shared leadership was also negative (*r* = −0.047, *P* < 0.001), indicating that the reasons for missed nursing care decrease as shared leadership increases. Furthermore, a strong positive correlation (*r* = 0.193, *P* < 0.001) has been seen between the elements of nursing care missed and the reasons for it.

## 4 Discussion

Shared leadership can significantly enhance interconnected working. Team members guide one another toward accomplishing a healthcare organization, group, or objectives through mutual influence and shared responsibility [23].

The results of the current study suggest that the nurses studied had a high-level perception of shared leadership. Additionally, there is a high level of perception in all dimensions. This result may be because the staff nurses collaborate regularly and trust each other. Additionally, they shared goals, values, and beliefs, and all were involved in the policymaking process. Also, a strong culture that promotes and values shared leadership practices among the nursing staff. This could be reflected in the organization’s policies, leadership development programs, and overall emphasis on collaborative decision-making. Nurses attend consistent team meetings, share goal-setting, and the delegation of leadership tasks and responsibilities among team members. The nurses may have had positive experiences with shared leadership, such as feeling empowered and supported, as the nurses have directly witnessed and benefited from this leadership approach. Moreover, the distinct proficiencies of every team member are appreciated and employed. According to Pendidikan, there was a high level of shared leadership [24].

In keeping with this, the study, which was carried out in Swedish intensive care units, revealed favorable perceptions of the characteristics that the staff had described. Shared leadership improves patient care by influencing the daily operations of different health professionals at the managerial and team levels through work motivation and confidence [25]. Additionally, a different study reveals that cultural characteristics were associated with the greatest mean percentage score of the nurses’ shared leadership style [26].

In addition, the studied nurses highly perceived the elements of missed nursing care. Furthermore, secondary care had the highest mean score, indicating patient education regarding discharge, oral care, and monitoring the effects of medication, all of which were deemed secondary care and were, therefore, greatly neglected. This could be identified by several potential causes, such as problems with workload and staffing when nurses are overworked in primary care and lack the time or resources to devote themselves fully to secondary care duties. High patient-to-nurse ratios or understaffing may cause nurses to prioritize urgent, necessary tasks above secondary care duties. Also, due to a lack of prioritization when faced with conflicting demands, nurses may deprioritize secondary care responsibilities because they are thought to be less urgent or vital than primary care duties.

Nobahar et al. provided support for this outcome by displaying that the dimension with the highest mean score of missed nursing care was “discharge planning and patient education,” while the dimension with the lowest mean score was “care intervention with ongoing assessment” [27]. On the other side, it moderated nurses’ perception of the reasons nurses missed care and showed the most common cause of missed nursing care was labor resources, which indicated low nurse staffing, low number of assistants and office staff, inappropriate rate of patient-to-nurse, unavailability of drugs when needed and material resource, and unavailability of required devices or dysfunctionality of devices as needed. All of these were the most common reasons for missed nursing care. This result aligns with prior research showing that hospital unit staffing and insufficient material resources are precursors to missed nurse care [28].

This study’s results showed statistical differences in the total score of the shared leadership based on all sociodemographic characteristics (*P* < 0.001). One way to justify this would be to assume that older nurses might be more exposed to various leadership experiences and styles, which could affect how they view and accept shared leadership practices within their teams. Also, the perception and implementation of shared leadership in nursing settings may be impacted by gender dynamics, which can influence leadership methods and communication styles. A nurse’s work-life balance and support system may be impacted by their marital status, which may also affect their participation in collaborative practices and shared leadership responsibilities. Higher-educated nurses might be more knowledgeable about modern leadership theories and practices, which could increase their understanding of shared leadership models. Nurses with more experience are probably more aware of team dynamics and might be more open to shared leadership, acknowledging its benefits in enhancing collaborative care.

Also, there were statistically differences in the total score of the elements of missed nursing care (part A of the MISSCARE survey) based on nurses’ sociodemographic characteristics, namely marital status, educational qualifications, and years of experience in the nursing profession. This could be rationalized by the assumption that nurses’ marital status may impact their support network and workload, which may hinder their capacity to handle duties and result in delayed care because of conflicting personal commitments. Furthermore, better training and skills are frequently associated with higher educational credentials, which help nurses detect and handle missing care more successfully, which affects their total care delivery. Finally, compared to nurses’ less experienced colleagues, experienced nurses are typically more confident and have more practical knowledge, which might improve their ability to prioritize duties and reduce missed nursing care.

Shared leadership was higher among nurses aged from 20 to less than 30 years old and married nurses with a bachelor’s degree in nursing sciences (*P* < 0.001). Both elements of and reasons for missed nursing care were higher among female single nurses (*P* < 0.001). The reasons for missed nursing care were higher among nurses with 5 to less than 10 years of experience in the nursing profession (since graduation). To defend this, the researchers could presume that newer training that prioritizes communication and teamwork may help younger nurses become more receptive to shared leadership and better adapted to collaborative practices. Also, married nurses may feel more secure and supported, which enables them to participate more fully in collaborative care settings and shared leadership responsibilities. Nurses with bachelor’s degrees are frequently taught teamwork and leadership techniques, which allows them to participate in shared leadership activities effectively. Due to possible time limits and support deficiencies, single female nurses may experience particular difficulties juggling their personal and professional obligations, which may result in a higher number of reports of nursing care missed. Lastly, as nurses manage the complexities of their jobs, nurses in this experience range may face growing workloads and responsibilities, leading to greater rates of missed care.

Supporting this study, Ghanem Atalla et al. found that nurses under 30 showed greater levels of involvement in leadership and shared governance roles, indicating that age may favor collaborative practice participation [29]. The findings that single female nurses face greater difficulties in providing care are consistent with research showing that female nurses, especially those who are unmarried, report more missed nursing care because of their increased personal responsibilities and lack of support [30].

The study’s result demonstrated that there was a significant negative correlation between overall missed nursing care and shared leadership. The correlation between the reasons for missing nursing care and shared leadership was also negative. The substantial inverse relationship between total missing nursing care and shared leadership implies that missed care is decreased when shared leadership is applied successfully. Better communication, teamwork, and accountability among nursing staff are encouraged by shared leadership, which helps them respond to care needs faster. Additionally, nurses are more likely to put patient care first and make fewer mistakes when they feel empowered and supported in a shared leadership framework. On the other hand, a lack of shared leadership can make nurses feel alone or overburdened, which increases the number of lost care opportunities. This emphasizes how crucial it is to incorporate shared leadership techniques to improve patient outcomes and guarantee the provision of comprehensive care.

This result aligns with Nobahar et al. who found that effective teamwork and shared leadership among nurses significantly reduced missed nursing care incidents. The authors emphasized that when nurses collaborate and share leadership responsibilities, they are better equipped to meet patient needs, leading to fewer omissions in care [31]. On the other hand, Babaei et al. indicated that not all leadership philosophies are successful in minimizing nursing care missed. In contrast to the advantages observed in shared leadership settings, a study found that typical hierarchical leadership models may increase the likelihood of missing care because of a lack of support and communication among nursing personnel [32].

By enabling the assessment of several variables in the population sample at one time, cross-sectional designs offer accurate data that are less prone to biases than case series or case reports. The study helps to understand shared leadership and missed nursing care from the perspective of nurses, but it also addresses a topic that has not received much scholarly attention in the healthcare environment. However, there are a few limitations to consider. First, because the study was restricted to a specific institution, the generalizability of the findings needs to be strengthened. Second, the study only examined the relationship between nurses’ thoughts of shared leadership and their perceptions of missed nursing care as a dependent variable, even if other variables that might impact missed nursing care were considered. A lot of data entry and cleaning was also required for a paper-based questionnaire.

## 5 Conclusions

This study concluded that the potential advantage of encouraging shared leadership cultures within healthcare institutions may result in more consistent and better use of nursing resources and necessary nursing care. The results of this study demonstrate the potential advantages of encouraging shared leadership cultures within healthcare institutions, as they may result in more consistent and better use of nursing resources and necessary nursing care. Hospital administrators should let nurse managers and nurses participate in educational and training programs, symposiums, conferences, and meetings on shared leadership practices and prevent causes of missed care in light of the results of the current study. Additionally, improving the lines of communication between nurse managers and nurses, facilitating the involvement of nurses in clinical decision-making and committees, and guaranteeing equitable resource distribution and opportunities are all important. Lastly, keep an eye on the organizational policies and encourage those that strengthen shared leadership practices and the incentive of nurses to stop missed nursing care. Nurse managers are crucial in fostering a positive work environment conducive to shared leadership and reducing missed care. Leadership support, promoting teamwork, and addressing staffing and resource issues are essential managerial strategies to improve patient care quality.

## Data availability statement

The datasets generated during and/or analyzed during the current study are available from the corresponding author upon reasonable request.

## CRediT authorship contribution statement

**Amal Diab Ghanem Atalla:** Conceptualization, Methodology, Validation, Formal analysis, Investigation, Data curation, Writing - original draft, Writing - review & editing, Project administration. **Naglaa Abdelaziz Mahmoud Elseesy:** Software, Validation, Writing - review & editing, Supervision. **Ayman Mohamed El-Ashry:** Conceptualization, Methodology, Data curation, Writing - review & editing, Supervision, Project administration. **Samia Mohamed Sobhi Mohamed:** Conceptualization, Methodology, Validation, Formal analysis, Investigation, Resources, Data curation, Writing - review & editing. **Ebaa Marwan Felemban:** Conceptualization, Methodology, Validation, Formal analysis, Investigation, Resources, Writing - review & editing. **Abdulhafith Alharbi:** Conceptualization, Methodology, Validation, Formal analysis, Investigation, Resources, Writing - review & editing. **Nervana Abdelrahman Saied Gheith:** Conceptualization, Methodology, Validation, Formal analysis, Writing - review & editing. **Sabrein Mahmoud khalifa khattab:** Conceptualization, Methodology, Validation, Formal analysis, Funding acquisition, Writing - review & editing.

## Declaration of competing interest

The authors have declared no conflict of interest.

## References

[1] Nasr A. (2018). Relation between shared governance and organizational commitment among nursing managers in port-said hospitals. Port Said Sci J Nurs.

[2] Bredelyte A., Skarbalius E. (2018). Proceedings of the international scientific conference.

[3] Van De Mieroop D., Clifton J., Verhelst A. (2020). Investigating the interplay between formal and informal leaders in a shared leadership configuration: a multimodal conversation analytical study. Hum Relat.

[4] Ropo A., Salovaara P. (2018). Spacing leadership as an embodied and performative process. Leadership.

[5] Zhu J.L., Liao Z.Y., Yam K.C., Johnson R.E. (2018). Shared leadership: a state-of-the-art review and future research agenda. J Organ Behav.

[6] Wu Q., Cormican K. (2020). Shared leadership and team effectiveness: an investigation of whether and when in engineering design teams. Front Psychol.

[7] Bragadóttir H., Kalisch B.J., Tryggvadóttir G.B. (2017). Correlates and predictors of missed nursing care in hospitals. J Clin Nurs.

[8] Chaboyer W., Harbeck E., Lee B.O., Grealish L. (2021). Missed nursing care: an overview of reviews. Kaohsiung J Med Sci.

[9] Beiboer C., Andela R., Hafsteinsdóttir T.B., Weldam S., Holtrop T., van der Cingel M. (2023). Teamwork, clinical leadership skills and environmental factors that influence missed nursing care - a qualitative study on hospital wards. Nurse Educ Pract.

[10] Kalisch B.J. (2006). Missed nursing care: a qualitative study. J Nurs Care Qual.

[11] Bassi E., Tartaglini D., Valpiani G., Grassetti L., Palese A. (2020). Unfinished nursing care survey: a development and validation study. J Nurs Manag.

[12] Iula A., Ialungo C., de Waure C., Raponi M., Burgazzoli M., Zega M. (2020). Quality of care: ecological study for the evaluation of completeness and accuracy in nursing assessment. Int J Environ Res Publ Health.

[13] Aiken L.H., Clarke S.P., Sloane D.M., Sochalski J.A., Busse R., Clarke H. (2001). Nurses' reports on hospital care in five countries. Health Aff.

[14] Molina-Mula J., Gallo-Estrada J. (2020). Impact of nurse-patient relationship on quality of care and patient autonomy in decision-making. Int J Environ Res Publ Health.

[15] Janatolmakan M., Khatony A. (2022). Explaining the consequences of missed nursing care from the perspective of nurses: a qualitative descriptive study in Iran. BMC Nurs.

[16] Arshared leadershipan G.G., Özden D., Göktuna G., Ertuğrul B. (2022). Missed nursing care and its relationship with perceived ethical leadership. Nurs Ethics.

[17] Dehghani F., Barkhordari-Sharifabad M., Fallah B., Khavari Z. (2023 Dec 10). Impact of ethical leadership on missed nursing care: a cross-sectional study from nurses' perspective. Avicenna Journal of Nursing and Midwifery Care.

[18] Elmedni B. (2015). Shared leadership or democratized decision-making process: practical lessons from Tunisia, Egypt and the USA. Int J Humanit Soc Sci.

[19] Forsyth C., Mason B. (2017). Shared leadership and group identification in healthcare: the leadership beliefs of clinicians working in interprofessional teams. J Interprof Care.

[20] Heyn L.G., Brembo E.A., Eide H., Hafskjold L., Sundling V. (2021). Older persons' expressed worries during nursing care at home: do health complexity and nature of nursing care in the visit matter?. Patient Educ Counsel.

[21] Brussow J.A. (2013).

[22] Kalisch B.J., Williams R.A. (2009). Development and psychometric testing of a tool to measure missed nursing care. J Nurs Adm.

[23] Hosseini Z., Raisi L., Maghari A., Karimollahi M. (2022 Jan 4). Translation and psychometric properties of the MISSCARE survey-Persian version. BMC Nurs.

[24] Ahmad N.H., Gani M.F. (2010). Analisis pelan induk pembangunan pendidikan (pipp) 2006-2010 digubal berdasarkan pembangunan pendidikan Malaysia (2001-2010). Jurnal International Manajemen Pendidikan.

[25] Rosengren K., Bondas T., Nordholm L., Nordström G. (2010). Nurses' views of shared leadership inICU: a case study. Intensive Crit Care Nurs.

[26] Ali Fouad Abd El Gwad Maged YH., Bassiouni N., Atalla A. (2021). Relationship between nursinggovernance and shared leadership style as perceived by nurses. International Journal of NovelResearch in Healthcare and Nursing.

[27] Nobahar M., Ameri M., Goli S. (2023). The relationship between teamwork, moral sensitivity, andmissed nursing care in intensive care unit nurses. BMC Nurs.

[28] Jones T.L., Hamilton P., Murry N. (2015). Unfinished nursing care, missed care, and implicitlyrationed care: state of the science review. Int J Nurs Stud.

[29] Ghanem Atalla A.D., Sharif L.S., Katooa N.E., Kandil F.S., Mahsoon A., Mahmoud Elseesy N.A. (2023). Relationship between nurses' perception of professional shared governance and their careermotivation: a cross-sectional study. Int J Nurs Sci.

[30] Alsadaan N., Salameh B., Reshia F.A.A.E., Alruwaili R.F., Alruwaili M., Awad Ali S.A. (2023). Impact of nurse leaders behaviors on nursing staff performance: a systematic review of literature. Inquiry.

[31] Nobahar M., Ameri M., Goli S. (2023). The relationship between teamwork, moral sensitivity, and missed nursing care in intensive care unit nurses. BMC Nurs.

[32] Babaei S., Amini K., Ramezani-Badr F. (2024). Unveiling missed nursing care: a comprehensive examination of neglected responsibilities and practice environment challenges. BMC Health Serv Res.

