# Supplementary material for: Unveiling the hidden connection: Investigating the relationship between shared leadership and missed nursing care
Source: Int J Nurs Sci. 2024 Dec 17;12(1):12–8. doi: 10.1016/j.ijnss.2024.12.012 (PMC11846603; doi:10.1016/j.ijnss.2024.12.012)
Supplement: Multimedia component 1 [file mmc1.docx]

埃及护士共享领导与护理缺失现状的调查与分析研究

Amal Diab Ghanem Atalla, Naglaa Abdelaziz Mahmoud Elseesy,Ayman Mohamed El-Ashry,

Samia Mohamed Sobhi Mohamed, Ebaa Marwan Felemban, Abdulhafith Alharbi, Nervana Abdelrahman Saied Gheith,Sabrein Mahmoud khalifa khattab

【摘要】

**目的** 该研究旨在探讨埃及护士共同领导及护理缺失的现状，并分析其相关性。

**方法** 采用横断面调查法。于2024年2月至4月，采用方便取样法选取亚历山大大学附属医院住院病房340 名护士为研究对象。采用共享领导问卷（Shared Leadership Questionnaire）和护理缺失量表（Missed Nursing Care Survey）进行调查。

**结果** 护士共享领导问卷得分为（72.62 ± 4.30）分（量表得分范围为20～100），表明护士共享领导总体水平较高。其中授权维度的均分最高为（3.66 ± 0.26）分；其次是合作维度为（3.64 ± 0.31）分；愿景维度均分最低为（3.59 ± 0.33）分。护理缺失量表中，护理缺失频率得分为（87.44 ± 7.97）分(得分范围为（24～120分），其中二级护理缺失均分较高，为（3.74 ± 0.31）分；护理缺失原因得分为（54.05 ± 3.42）分（得分范围为17～68分），其中人力资源原因均分较高为（3.20 ± 0.22）分。男性、30岁以下、已婚、本科学历、护理经验及在目前岗位工作均少于5年的护士共享领导力得分较高，护理缺失量表得分较低(*P* < 0.001)。相关分析结果显示，共享领导力得分与护理缺失类型（*r* = ﹣0.383）及护理缺失原因（*r* = ﹣0.047）2个维度得分均呈负相关（*P* < 0.001）。

**结论** 护理领导者可通过鼓励护士的团队合作，提高其责任感、工作量管理及授权，为减少护理差错的发生提供有利环境。

【关键词】护理缺失；护理人员；护理管理研究；共享领导

通信作者：Amal Diab Ghanem Atalla, E-mail: [aml.diab@alexu.edu.eg](mailto:aml.diab@alexu.edu.eg)
